# Oligodendrocyte Precursor Cells Support Blood-Brain Barrier Integrity via TGF-β Signaling

**DOI:** 10.1371/journal.pone.0103174

**Published:** 2014-07-31

**Authors:** Ji Hae Seo, Takakuni Maki, Mitsuyo Maeda, Nobukazu Miyamoto, Anna C. Liang, Kazuhide Hayakawa, Loc-Duyen D. Pham, Fumihiko Suwa, Akihiko Taguchi, Tomohiro Matsuyama, Masafumi Ihara, Kyu-Won Kim, Eng H. Lo, Ken Arai

**Affiliations:** 1 Neuroprotection Research Laboratory, Departments of Radiology and Neurology, Massachusetts General Hospital and Harvard Medical School, Charlestown, Massachusetts, United States of America; 2 SNU-Harvard NeuroVascular Protection Research Center, College of Pharmacy and Research Institute of Pharmaceutical Sciences, Seoul National University, Seoul, Korea; 3 Department of Anatomy, Osaka Dental University, Osaka, Japan; 4 Department of Regenerative Medicine, Institute of Biomedical Research and Innovation, Kobe, Japan; 5 Laboratory of Neurogenesis and CNS Repair, Institute for Advanced Medical Science, Hyogo College of Medicine, Hyogo, Japan; 6 Department of Stroke and Cerebrovascular Diseases, National Cerebral and Cardiovascular Center, Osaka, Japan; 7 Department of Molecular Medicine and Biopharmaceutical Sciences, Graduate School of Convergence Science and Technology, and College of Medicine or College of Pharmacy, Seoul National University, Seoul, Korea; University of South Florida, United States of America

## Abstract

Trophic coupling between cerebral endothelium and their neighboring cells is required for the development and maintenance of blood-brain barrier (BBB) function. Here we report that oligodendrocyte precursor cells (OPCs) secrete soluble factor TGF-β1 to support BBB integrity. Firstly, we prepared conditioned media from OPC cultures and added them to cerebral endothelial cultures. Our pharmacological experiments showed that OPC-conditioned media increased expressions of tight-junction proteins and decreased in vitro BBB permeability by activating TGB-β-receptor-MEK/ERK signaling pathway. Secondly, our immuno-electron microscopic observation revealed that in neonatal mouse brains, OPCs attach to cerebral endothelial cells via basal lamina. And finally, we developed a novel transgenic mouse line that TGF-β1 is knocked down specifically in OPCs. Neonates of these OPC-specific TGF-β1 deficient mice (OPC-specific TGF-β1 partial KO mice: *Pdgfra^Cre^*/*Tgfb1^flox/wt^* mice or OPC-specific TGF-β1 total KO mice: *Pdgfra^Cre^*/*Tgfb1^flox/flox^* mice) exhibited cerebral hemorrhage and loss of BBB function. Taken together, our current study demonstrates that OPCs increase BBB tightness by upregulating tight junction proteins via TGF-β signaling. Although astrocytes and pericytes are well-known regulators of BBB maturation and maintenance, these findings indicate that OPCs also play a pivotal role in promoting BBB integrity.

## Introduction

The concept of the neurovascular unit is now relatively well accepted. The neurovascular unit emphasizes that cell-cell interaction between neuronal, glial, and vascular elements is critical for brain function [1]. One of the most documented non-cell autonomous phenomena in the neurovascular unit is the blood-brain barrier (BBB). BBB is an essential property of brain blood vessels that allow the brain to maintain its highly specialized microenvironment and homeostasis [2], [3]. BBB function is sustained by trophic coupling between cerebral endothelium and neighboring cells, such as pericytes and astrocytes [4], [5], [6].

Trophic coupling of endothelium-pericyte-astrocyte tightly regulates BBB formation and maturation [7]. According to current understanding, pericytes are recruited to cerebral endothelial cells to initiate BBB properties during embryogenesis. Cerebral endothelial cells release platelet-derived growth factor (PDGF) to recruit pericytes, which express PDGF-receptor-β [8]. Adhesion between cerebral endothelial cells and pericytes is mediated by transforming growth factor β (TGF-β) secreted from the cell types in paracrine and autocrine manners. TGF-β stimulates the production of extracellular matrix in pericytes, which then induces the upregulation of cadherin-2 in cerebral endothelial cells [5], [9]. After pericytes are set in place, astrocytes are subsequently recruited to enhance BBB tightness during development [8] and maintain BBB function [10]. Angiopoietin 1 and Sonic hedgehog secreted from astrocytes increase tight junction protein expression and barrier function of cerebral endothelial cells [11], [12]. Astrocytes also produce apolipoprotein E to promote BBB maturation through lipoprotein receptor-related protein in cerebral endothelial cells [13], [14].

However, besides astrocytes and pericytes, other types of brain cells also exist in the brain. Our recent studies have proposed that oligodendrocyte lineage cells may communicate with cerebral endothelial cells via the exchange of soluble factors [15], [16]. For example, trophic factors including fibroblast growth factor -2 (FGF-2) and brain-derived neurotrphic factor (BDNF) from cerebral endothelial cells may promote the proliferation and survival of oligodendrocyte precursor cells (OPCs) even under pathologic conditions [17]. In addition, cerebral endothelial cells may also support OPC migration via secreting vascular endothelial growth factor [18]. In turn, oligodendrocyte lineage cells may participate in vascular dysfunction and repair after white matter injury [15], [19]. Since oligodendrocyte precursor cells can act as a critical source of trophic factors [20], it may be possible that OPCs also help regulate BBB integrity. Therefore, in this study, we explore the hypothesis that trophic coupling between cerebral endothelium and OPCs is fundamentally BBB-protective.

## Materials and Methods

### Cell Culture

All experiments were reviewed and approved by a Subcommittee for Research Animal Care of the Massachusetts General Hospital IACUC (Institutional Animal Care and Use Committee) and all these institutionally-approved animal protocols are consistent with the NIH Guide for the Care and Use of Laboratory Animals. OPCs were prepared from cerebral cortices of 1–2 day old Sprague-Dawley (SD) rats. Dissociated cortex cells were plated in poly-d-lysine-coated flasks, and culture in Dulbecco’s Modified Eagle’s medium containing 20% fetal bovine serum and 1% penicillin/streptomycin. After the cells were confluent, the flasks were shaken for 1 hour on an orbital shaker (220 rpm) at 37°C to remove microglia. The medium was changed with a new medium and shaken overnight. The medium was then collected and plated on non-coated tissue culture dishes for 1 hour at 37°C to eliminate possible contamination by astrocytes and microglia. The non-adherent cells were collected and cultured in Neurobasal Media containing glutamine, 1% penicillin/streptomycin, 10 ng/mL PDGF-AA, 10 ng/mL FGF-2, and 2% B27 supplement onto poly-dl-ornithine-coated plates. Rat brain microendothelial cell lines (RBE.4) were cultured in EBM-2 containing EGM-2MV Single Quots kit onto collagen-coated flasks.

### Preparation OPC conditioned medium

OPC cultures were washed with PBS and maintained in Neurobasal media for 24 hours. Culture medium was then collected and centrifuged at 10,000 g for 5 min at 4°C to remove cells and debris. The OPC conditioned medium was stored at −80°C until use.

### Immunocytochemistry

After cells were confluent, they were washed with ice-cold PBS (pH 7.4), followed by 4% PFA for 15 min. After being further washed three times in PBS, they were incubated with 3% BSA in PBS for 1 hour. Cells were then incubated with primary antibody against ZO-1 (1∶200, Invitrogen) or Smad2/3 (1∶200, Santacruz) at 4°C overnight. After washing with PBS, they were incubated with secondary antibodies conjugated with fluorescein isothiocyanate for 1 hour at room temperature. Finally, nuclei were counterstained with DAPI. Image was analyzed with a fluorescence microscope (Nikon) interfaced with a digital charge-coupled device camera and an image analysis system.

### In vitro endothelial permeability assay

Permeability across the endothelial cell monolayer was measured by using type I collagen-coated transwell units (6.5 mm diameter, 3.0 µm pore size polycarbonate filter, Corning). After RBE.4 cells became confluent on the transwell, the cells were treated with OPC conditioned media for 24 hours. Then, fluorescein isothiocyanate-labeled dextran (molecular weight, 40,000) was added to the upper chamber. After incubation for 10 min, 100 µl of sample from the lower compartment was measured for fluorescence at 620 nm when excited at 590 nm with a spectrophotometer.

### Cell viability assay

To ensure that OPC-conditioned media has no effect on endothelial cells survival, cell viability was quantified by lactate dehydrogenase (LDH) release with the use of the LDH assays kit (Roche).

### Immunohistochemistry

Mouse brains were taken out from 0–1 day old pups. After perfusion with PBS (pH 7.4), brains were quickly frozen using powdered dry ice. Coronal sections of 16-µm thicknesses were cut on cryostat at −20°C and collected on glass slides. Sections were fixed by 4% PFA, and rinsed three times in PBS (pH 7.4). After blocking with 3% BSA, sections were incubated at 4°C overnight in PBS/0.1% Tween/0.3% BSA solution containing primary antibodies –anti-NG2 (a marker for OPC, 1∶100, Millipore), anti-PDGF-receptor-α [CD140a] (a marker for OPC, 1∶100, Santacruz), anti-PDGF-receptor-β [CD140b] (a marker for pericyte, 1∶100, eBioscience), anti-GFAP (a marker for astrocyte, 1∶200, BD Pharmingen), anti-CD31 (a marker for endothelial cell, 1∶100, BD Pharmingen), anti-Cre (1∶100, Millipore), and anti-TGF-β (1∶100, Abcam). Then sections were washed and incubated with secondary antibodies with fluorescence conjugations at room temperature for 1 hr. For lectin staining, sections were incubated in fluorescein labeled Lycopersicon Esculentum (Tomato) Lectin (1∶200, Vector Laboratories) at room temperature for 15 min. Subsequently, sections were washed and the slides were covered with VECTASHIELD mounting medium with DAPI (H-1200 from Vector Laboratories). Immunostaining was analyzed with confocal microscopy or fluorescence microscope (Nikon) interfaced with a digital charge-coupled device camera and an image analysis system.

### Immuno-Electron Microscopy

For electron microscopic investigation of PDGF-receptor-α, postnatal day 1–2 SD rats were perfused trans-cardially with 0.1% glutaraldehyde-4% PFA fixative, and brains were immersed in the 4% PFA at 4°C for 5 h and sectioned at a thickness of 50 µm with a microslicer (Dosaka EM). After blocking with 10% NGS, sections were incubated with anti- PDGF-receptor-α (1:100, Santa Cruz, CA, USA) at 4°C overnight. They were then incubated with biotinylated anti-rabbit IgG (1:200), Vectastain ABC reagent (Vector Laboratories, Inc., Burlingame, CA) according to the manufactur’s procedures and visualized by 3, 3′-diaminobenzidine tetrahydrochloride-H_2_O_2_ solution. After the DAB reaction, sections were post-fixed with 1% OsO4 for 1 h, dehydrated, and flat-embedded on siliconized glass slides in Epon. Ultrathin sections from corpus callosum were stained with 2% uranyl acetate and lead citrate. Electron micrographs were taken at 80 kV on a JEM-1200EX electron microscope.

### Breeding and Genotyping of Mutant mice

*Pdgfra^Cre^* mice and *Tgfb1^flox/flox^* mice were purchased from Jackson Laboratories. *Pdgfra^Cre^* mice with a C57BL/6 genetic background are a strain of mice expressing Cre recombinase under the control of a Pdgfra (the gene for platelet-derived growth factor receptor-alpha) genomic DNA fragment. *Tgfb1^flox/flox^* mice in a mixed 129×C57BL/6 genetic background are a strain of mice carrying a loxP-flanked *Tgfb1* gene. First, *Pdgfra^Cre^* mice were crossed with Tgf-beta1lox/lox mice. Subsequently, *Pdgfra^Cre^*/*Tgfb1^flox/wt^* double heterozygotes were bred to *Tgfb1^flox/flox^* mice. Genotyping was done by PCR analysis using genomic DNA from mouse tail biopsies. Genotyping primers for the detection of *Pdgfra^Cre^* transgene (product size ∼100 bp) were 5′-GCG GTC TGG CAG TAA AAA CTA TC-3′ (forward) and 5′-GTG AAA CAG CAT TGC TGT CAC TT-3′ (reverse). Genotyping primers for the detection of Tgfb1 wild-type (product size 216 bp) and loxP-flanked alleles (277 bp) were 5′-AAG ACC TGG GTT GGA AGT G-3′ (forward) and 5′-CTT CTC CGT TTC TCT GTC ACC CTA T-3′ (reverse).

### Western Blotting

Lysates from the corpus callosum region were prepared in Pro-PREPTM Protein Extraction Solution (Boca Scientific). Samples with equal volumes of SDS sample buffer (Novex) and 2-ME were heated at 95°C for 5 min, then each sample (20 µg per lane) was loaded onto 4–20% Tris–glycine gels. After electrophoresis and transferring to polyvinylidene difluoride membranes (Novex), the membranes were blocked in Brockace (AbD Serotec) for 60 min at room temperature. Membranes were then incubated overnight at 4°C with anti-ZO-1 antibody (1∶1000, Invitrogen), anti-occludin antibody (1∶1000, Abcam), anti-claudin5 (1∶1000, Abcam), anti-ERK1/2 (1∶1000, Cell Signaling), anti-phospho-ERK1/2 (1∶000, Cell Signaling) or anti-β-actin antibody (1∶5000, Sigma Aldrich) followed by incubation with peroxidase-conjugated secondary antibodies and visualization by enhanced chemiluminescence (Amersham).

### IgG staining

Mouse brains were taken out after perfusion with 0.9% saline and quickly frozen using powdered dry ice. Coronal sections of 20µm thickness were cut on cryostat at −20°C and collected on glass slides. Sections were fixed by 4% PFA and rinsed 3 times in PBS (pH 7.4). Subsequently, the sections were incubated in 3% H_2_O_2_, followed by blocking with 10% Brockace (AbD serotec) in PBS. Then the sections were incubated overnight at 4°C with antibody against donkey anti-mouse IgG (1∶300; Jackson Immunoresearch Laboratories). Immunoreactivity was visualized using fluorescence-conjugated streptavidin. Staining was analyzed with a fluorescence microscope (Nikon) interfaced with a digital charge-coupled device camera and an image analysis system. The acquired images were processed using Adobe Photoshop (version 7, Adobe System). RGB images were converted to 8-bit grayscale images. Based on an analysis of pixel fluorescence intensities, which ranged from 0 to 255, specific staining was distinguished from background by using a threshold value of 40–50. Area densities of structures stained with IgG were calculated as the proportion of pixels having a fluorescence intensity value equal to or greater than the threshold. Fields in the cortex were examined and IgG staining area was calculated by determining the number of pixels with positive staining per microscope field: this ratio was expressed as % area.

### Statistical Analysis

Statistical significance was evaluated using the unpaired t-test to compare differences between the two groups and a one-way ANOVA followed by Tukey’s honestly significant difference test for multiple comparisons. Data are expressed as mean ± S.D. A p-value of <0.05 was considered statistically significant.

## Results

**Figure 1:**
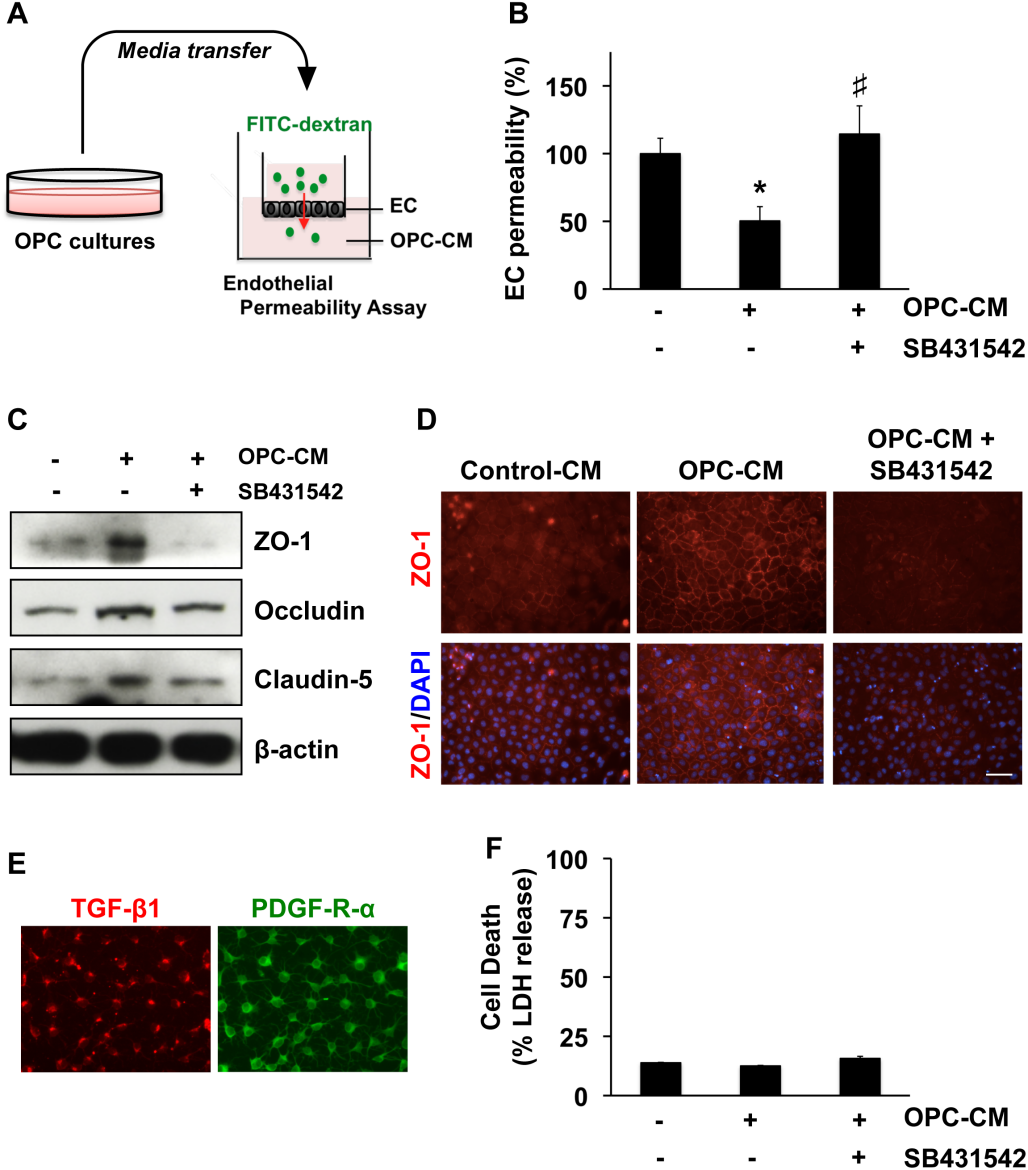
OPC-derived TGF-β1 and BBB integrity in vitro. **A.** We prepared conditioned media from OPCs (OPC-CM), and then added them to cerebral endothelial RBE.4 cells. **B.** OPC-CM increased in vitro BBB tightness (i.e. decreased permeability of endothelial monolayer), and the TGF-β-receptor signaling inhibitor SB431542 (10 µM) cancelled the effects of OPC-CM. Mean ± SD of n = 3. *p<0.05 vs control (OPC-CM: −, SB431542: −), #P<0.05 vs OPC-CM only (OPC-CM: +, SB431542: −). **C–D.** Correspondingly, OPC-CM increased the expressions of tight junction proteins, and once again, SB431542 (10 µM) cancelled the effects of OPC-CM. **E.** Immunostaining confirmed that our cultured rat OPCs produced TGF-β1. PDGF-R-α staining was used to confirm the purity of our OPC cultures. **F.** LDH assay showed that the TGF-β receptor inhibitor SB431542 (10 µM) did not affect endothelial viability. Mean ± SD of n = 3.

We prepared primary OPC cultures from rat neonatal brain cortex. When conditioned media were collected from rat OPC cultures and added to rat cerebral endothelial RBE.4 cells, the dextran permeability of these endothelial monolayers was significantly decreased (Figure 1A–B). This response was accompanied by an upregulation in the endothelial tight junction proteins ZO-1, occludin and claudin-5 (Figure 1C–D). Tight junctions are known to be regulated by TGF-β1 [21]. So next we examined the role of TGF-β1 in our model system. OPCs expressed TGF-β1 in culture (Figure 1E), and co-treatment with the TGF-β receptor inhibitor SB431542 prevented OPC conditioned media from upregulating tight junction proteins and decreasing endothelial permeability (Figure 1B–E). The LDH assay showed that the SB431542 treatment did not affect the endothelial viability in our cell culture system (Figure 1F).

**Figure 2:**
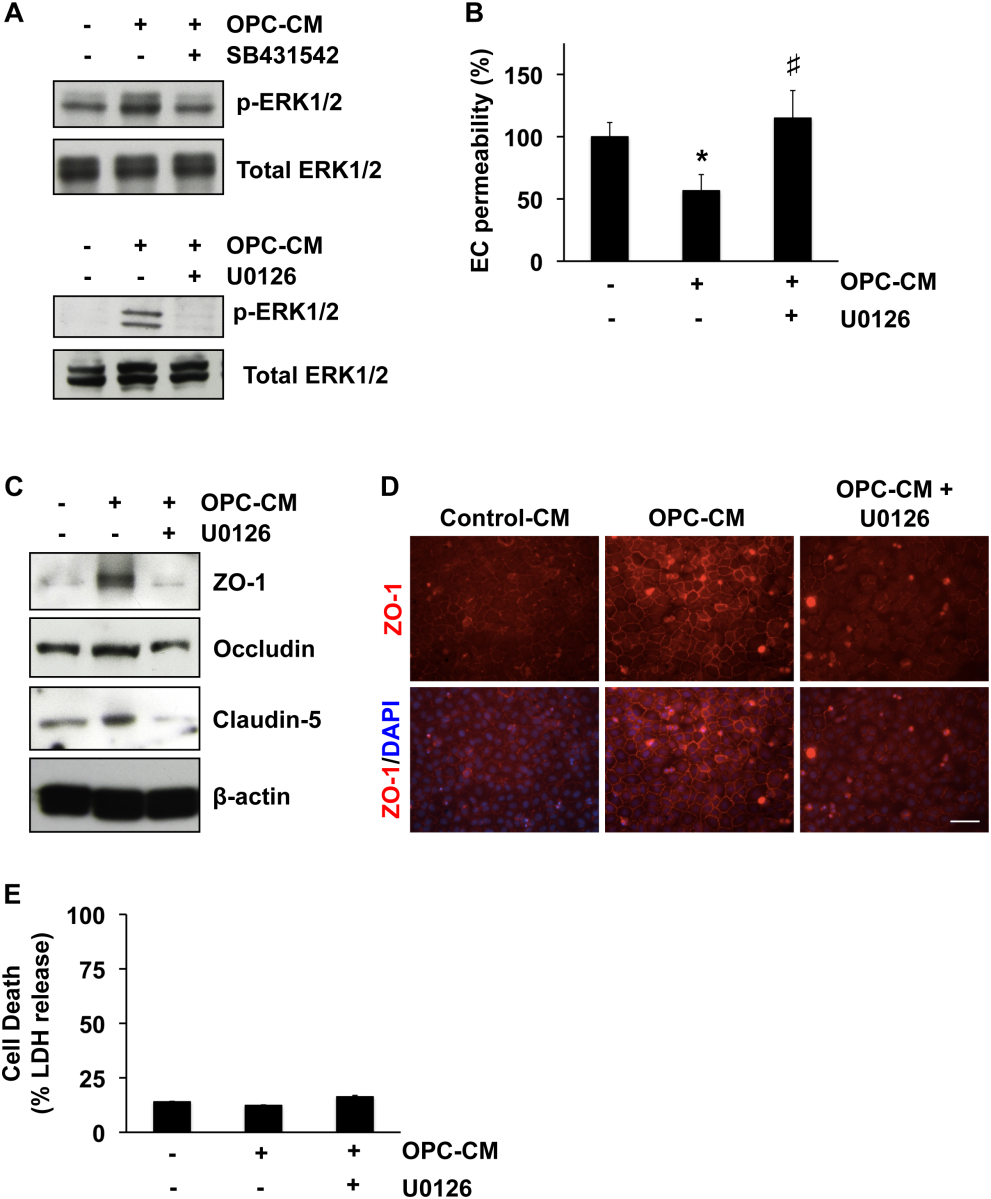
MEK/ERK pathway mediates the roles of OPC-derived TGF-β1 in enhancing in vitro BBB integrity. **A.** OPC-CM treatment (30 min) increased the levels of phospho-ERK1/2 in cerebral endothelial RBE.4 cells, and both the TGF-β receptor inhibitor SB431542 (10 µM) and a MEK/ERK inhibitor U0126 (10 µM) blocked the ERK1/2 phosphorylation by OPC-CM. **B–D.** U0126 cancelled the supporting effects of OPC-CM on the BBB tightness in our in vitro model. Mean + SD of n = 3, *p<0.05 vs control (OPC-CM: −, U0126: −), #P<0.05 vs OPC-CM only (OPC-CM: +, U0126: −). **E.** LDH assay showed that the MEK/ERK inhibitor U0126 (10 µM) did not affect endothelial viability. Mean ± SD of n = 3.

Our pharmacological experiments also identified that MEK/ERK pathway mediates the enhancement of in vitro endothelial tightness by OPC-derived TGF-β1. Conditioned media from OPC cultures increased the level of ERK1/2 phosphorylation, which was blocked by co-treatment with SB431542 or the MEK/ERK inhibitor U0126 (Figure 2A). Correspondingly, U0126 cancelled the supportive effects of OPC-conditioned media on cerebral endothelial cells - endothelial permeability (Figure 2B) and tight junction protein expression (Figure 2C–D). Again, the LDH assay confirmed that the U0126 treatment did not induce overt cell death in the endothelial cultures (Figure 2E). Taken together, these data suggest that OPCs secrete TGF-β1 to support cerebral endothelial barrier function in vitro.

**Figure 3:**
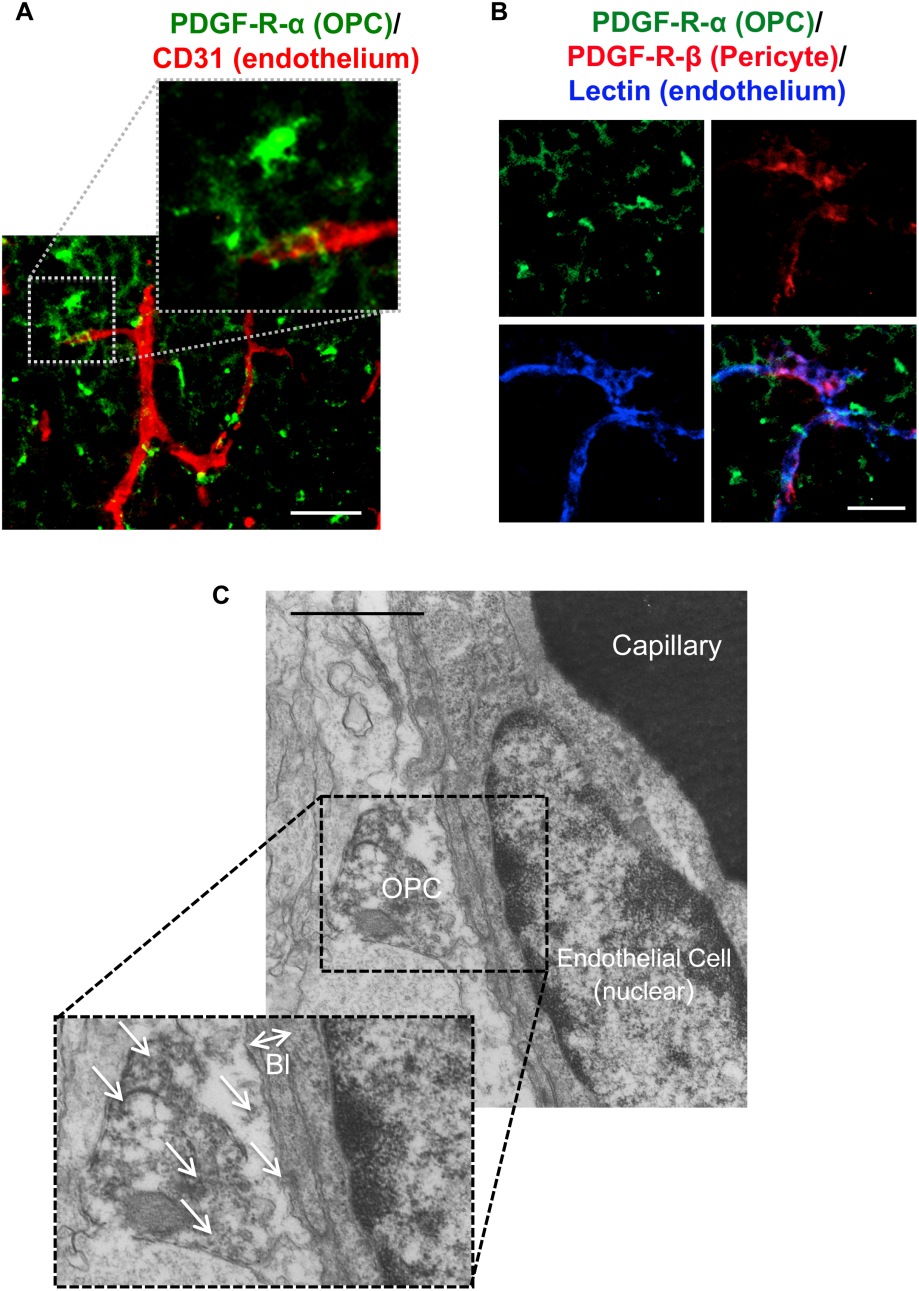
OPC-endothelium interaction. **A.** Immunostaining showed that some OPCs (green: PDGF-R-α) are located closely to cerebral endothelium (red: CD31) in mice at post-natal day 0–1. Scale bar = 50 µm. **B.** Triple staining showed that there is little overlap between PDGF-R-α and PDGF-R-β. Scale bar = 10 µm. **C.** Electron micrography also confirmed that OPCs attached directly to the basal lamina (Bl) of endothelial cells in corpus callosum. arrows: PDGF-R-α positive signals, Scale bar = 1 µm.

Since OPCs can increase barrier tightness in cerebral endothelial cell cultures, we then asked whether this phenomenon may occur in vivo. Immunostaining of brain sections from postnatal mice showed that micro-vessels (CD31, red color) were wrapped with fine processes emanating from OPCs (PDGF-receptor-α, green color) (Figure 3A). Triple staining with lectin (blue color, endothelial cell marker), PDGF-receptor-β (red color, pericyte marker), and PDGF-receptor-α (green color, OPC marker) showed that at least around the blood vessels, PDGF-receptor-α was indeed a specific OPC marker, i.e. there was no overlap between PDGF-receptor-β (pericytes) and PDGF-receptor-α (OPCs) (Figure 3B). To further confirm that OPCs are closely located to cerebral endothelium, we then conducted immuno-electron microscopic analysis using anti-PDGF-receptor-α antibody. Again, PDGF-receptor-α-positive OPCs were observed to attach to cerebral endothelium via basal lamina structures (Figure 3C).

**Figure 4:**
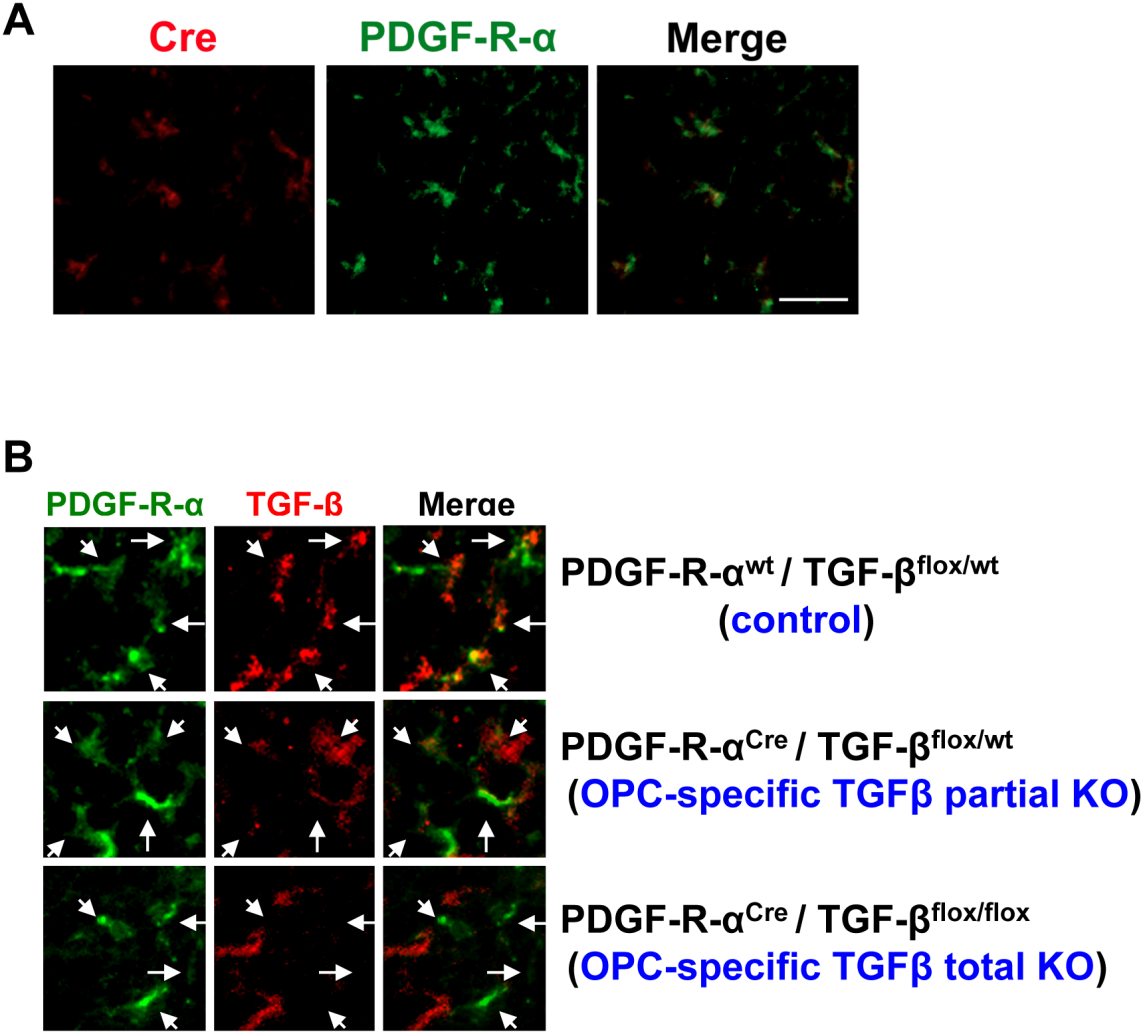
OPC-specific TGF-β1 deficient mice. **A.** Immunostaining showed that in *Pdgfra^Cre^* mice, Cre recombinase expression was observed only in PDGF-R-α positive cells (e.g. OPCs) at post-natal day 0. Scale bar = 25 µm. **B.** Compared to wild-type mice (*Pdgfra^wt^*/*Tgfb1^flox/wt^*), TGF-β1 expression in OPCs at post-natal day 0 was reduced or absent in OPC-specific TGF-β1 partial KO mice (*Pdgfra^Cre^*/*Tgfb1^flox/wt^* mice) or OPC-specific TGF-β1 total KO mice (*Pdgfra^Cre^*/*Tgfb1^flox/flox^* mice), respectively. Arrows indicate PDGF-R-α positive cells (e.g. OPCs).

**Figure 5:**
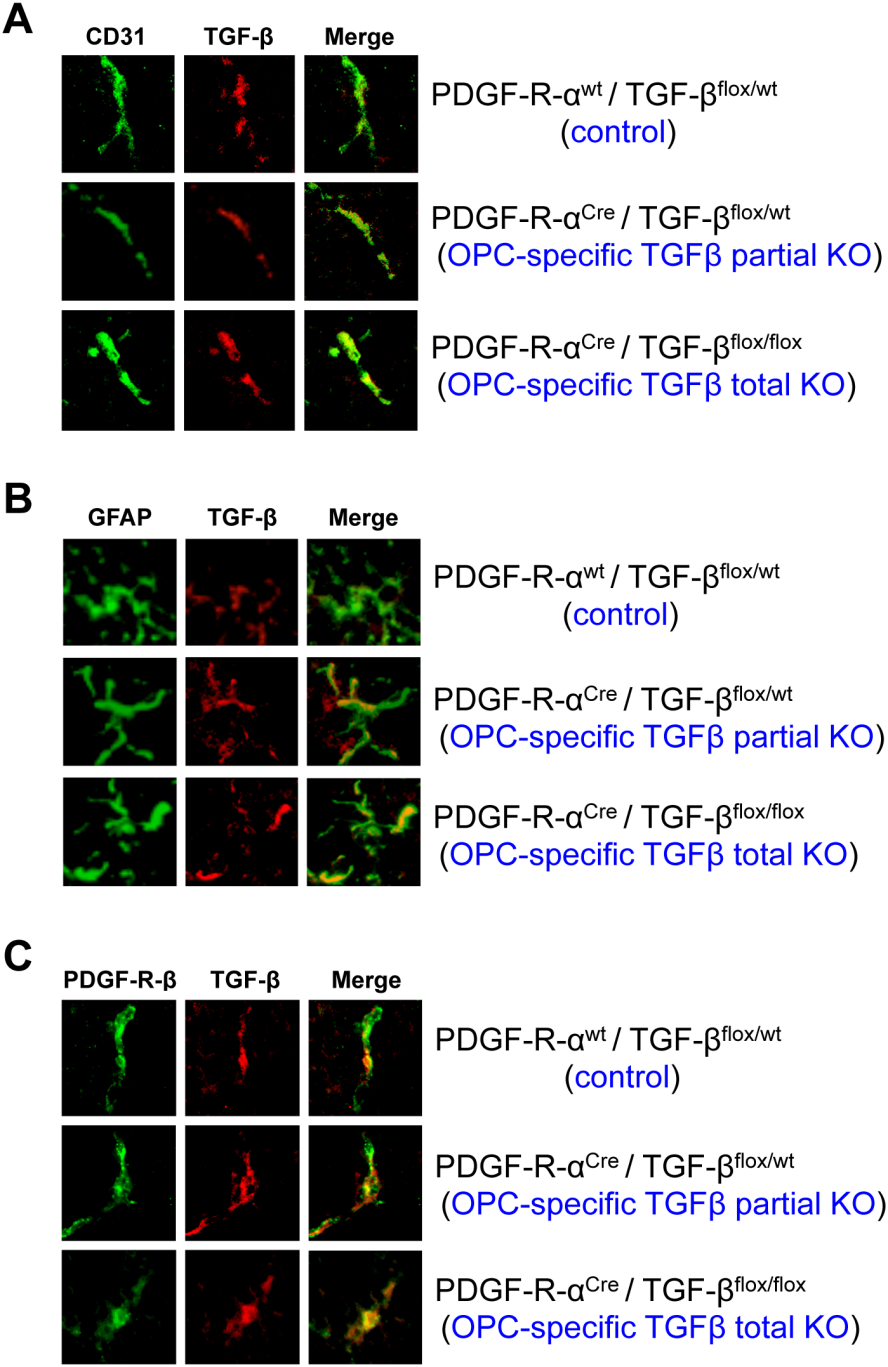
TGF-β1 expression in vascular-related brain cells. Immunostaining confirmed no clear differences in TGF-β1 expression in (**A**) cerebral endothelium assessed by CD31 staining, (**B**) astrocytes assessed by GFAP staining, and (**C**) pericytes assessed by PDGF-R-β staining at post-natal day 0 in the OPC-specific TGF-β1 deficient mice (OPC-specific TGF-β1 partial KO mice: *Pdgfra^Cre^*/*Tgfb1^flox/wt^* mice or OPC-specific TGF-β1 total KO mice: *Pdgfra^Cre^*/*Tgfb1^flox/flox^* mice).

**Figure 6:**
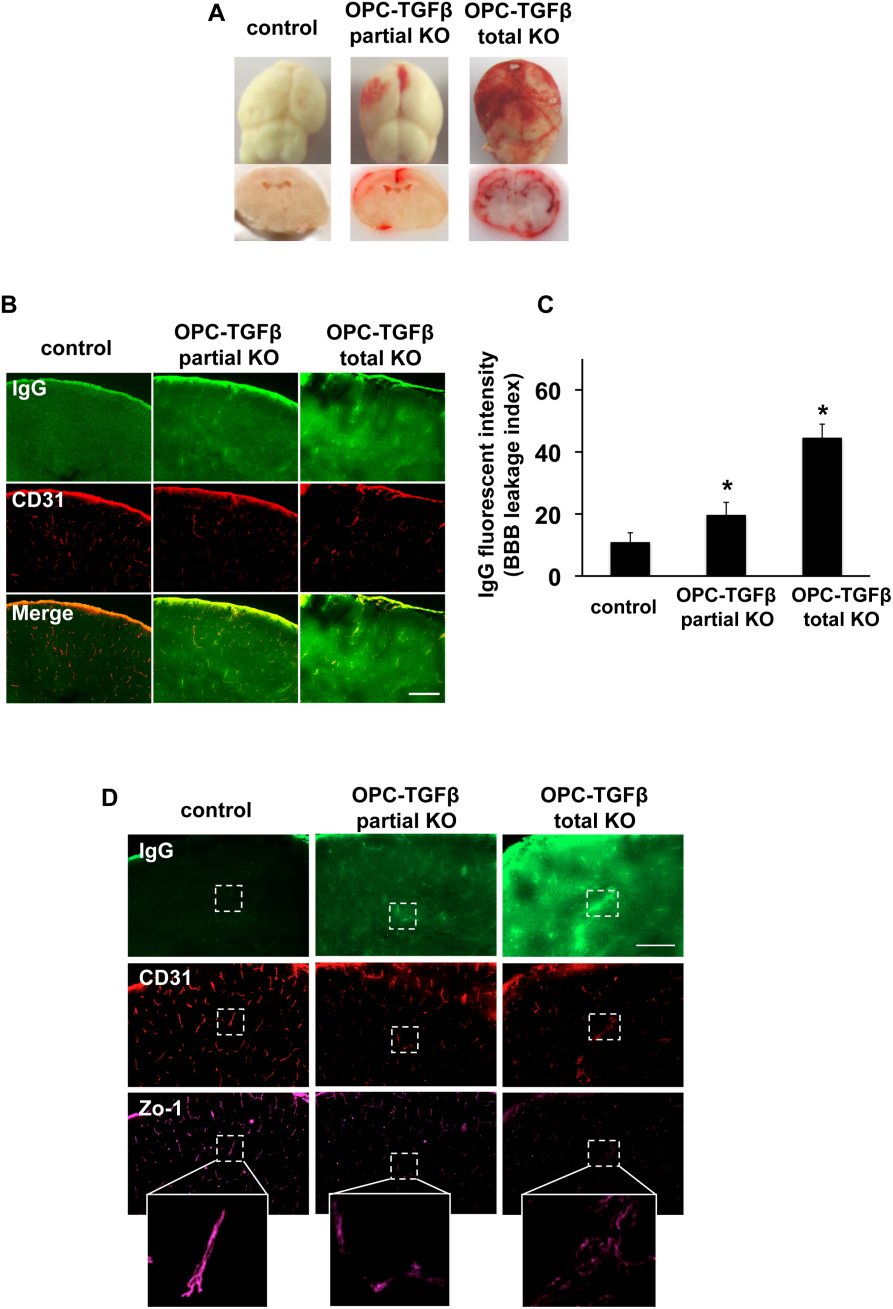
OPC-derived TGF-β1 and BBB integrity in vivo. **A.** The OPC-specific TGF-β1 partial or total KO mice showed hemorrhage at post-natal day 0–1. **B–C.** IgG staining showed that in the OPC-specific TGF-β1 partial or total KO mice, IgG was leaked from blood vessels into brain parenchyma. Green: IgG, Orange: Lectin (endothelial marker), scale bar = 100 µm. Mean + SD of n = 4–5, *p<0.05 vs control group. **D.** The OPC-specific TGF-β1 deficient mice exhibited BBB leakages (IgG staining) and aberrant ZO-1 structures.

Finally, we developed a line of transgenic mice with OPC-specific TGF-β1 deficiency using *Pdgfra^Cre^* mice that express Cre recombinase under the control of a Pdgfra (the gene for platelet-derived growth factor receptor-alpha) genomic DNA fragment. Cre staining confirmed that in the *Pdgfra^Cre^* mice, Cre expressions were restricted to PDGF-R-α-positive OPCs at post-natal day 0 (Figure 4A). OPC-specific TGF-β1 deficient mice were generated by crossing *Pdgfra^Cre^* mice with *Tgfb1^flox/flox^* mice that carry a loxP-flanked Tgfb1 gene. Immunostaining confirmed that TGF-β1 expression in OPCs was reduced or absent in *Pdgfra^Cre^*/*Tgfb1^flox/wt^* mice (OPC-specific TGF-β1 partial knockout (KO) mice) or *Pdgfra^Cre^*/*Tgfb1^ flox/flox^* mice (OPC-specific TGF-β1 total KO mice), respectively (Figure 4B). TGF-β1 expression was not affected in other vascular-related brain cells, such as cerebral endothelium, astrocytes or pericytes (Figure 5). Importantly, compared to control mice, OPC-specific TGF-β1 total KO mice showed massive cerebral hemorrhage, and even OPC-specific TGF-β1 partial KO mice exhibited cerebral hemorrhage to some extent at post-natal day 0–1 (Figure 6A). Staining for IgG leakage demonstrated that significant leakage of this large serum protein was observed in the OPC-specific TGF-β1 deficient mice (Figure 6B–C). Similar to the findings from our in vitro study, the tight junction protein ZO-1 was degraded in the transgenic mice (Figure 6D), suggesting that OPC-derived TGF-β1 plays an important role in BBB integrity.

## Discussion

**Figure 7:**
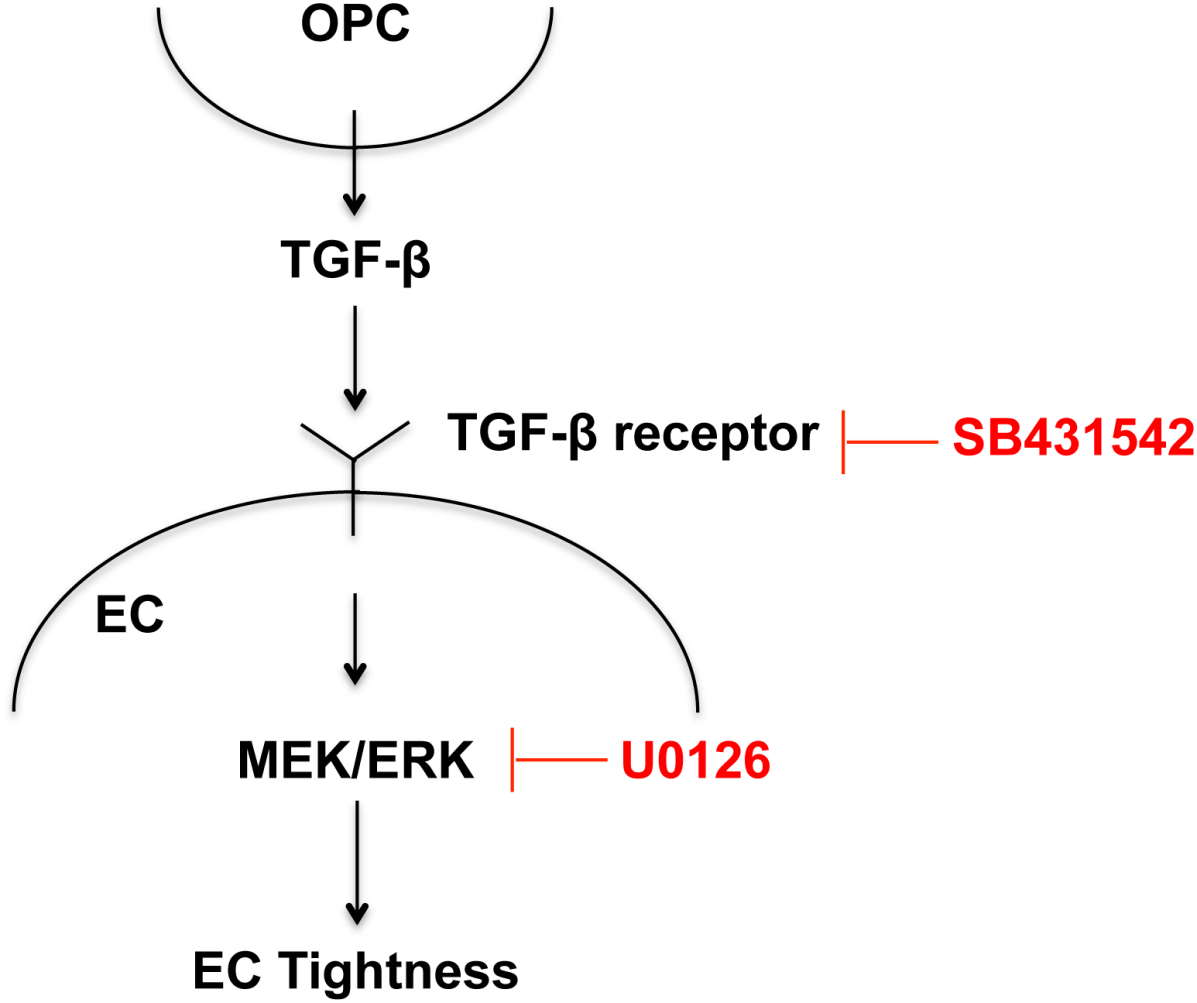
Proposed model for OPC-BBB interaction. Trophic coupling between OPCs and cerebral endothelium may play an important role in the BBB function. TGF-β1 may mediate the cell-cell interaction; i.e. OPCs secrete TGF-β1, which then binds to its receptor, followed by activating MEK/ERK pathway to increase the BBB tightness.

Our data demonstrate that OPCs are an important source of TGF-β1 that supports BBB integrity during development. OPC-derived TGF-β1 activates the MEK/ERK pathway in cerebral endothelium via TGF-β receptor, and the signaling cascade eventually increases the expression levels of tight-junction proteins to promote the BBB integrity. TGF-β1 is well known to modulate blood vessel development and maturation. For example, TGF-β1 KO mice had a severe deficiency in formation and maintenance of vasculatures, and TGF-β-receptor KO mice exhibited defective yolk sac vasculogenesis [22], [23], [24]. In addition, past cell culture studies have shown that astrocyte-endothelium or pericyte-endothelium trophic coupling induce the activation of TGF-β1 signaling, which is required for vascular barrier function [25], [26]. Our current study may expand the roles of TGF-β signaling in vascular system; i.e. OPCs can secrete TGF-β1 to support/enhance BBB tightness via a MEK/ERK signaling pathway (Figure 7).

Our present findings are significant because they imply that OPCs are not merely precursor cells for oligodendrocytes. Instead, OPCs might additionally participate in balancing brain homeostasis by secreting soluble factors. During development, progenitor cells in the subventricular zone (SVZ) serve as primary precursors for new oligodendrocytes and give rise to young migrating OPCs. These newborn cells move out of the SVZ into the corpus callosum, striatum, and fimbria fornix and then differentiate into myelinated oligodendrocytes. Beyond this expected role in brain development, our current study suggests a novel role for OPCs that they secrete soluble factors to regulate neighboring microenvironments. While little is known in the case of OPCs, mature oligodendrocytes are now well accepted to act as growth factor providers for neighboring cells [20], [27]. For example, oligodendrocytes synthesize defined growth factors and provide trophic signals to nearby neurons. Previous studies show that oligodendrocyte-derived trophic factors, such as insulin-like growth factor-1 and glial cell derived neurotrophic factor, promote neuronal survival and axonal outgrowth in vitro [28]. Moreover, oligodendrocytes may serve as a principal metabolic supplier of lactate, which is integral for axonal energy support through monocarboxylate transporter 1 [29]. Therefore, similar to mature oligodendrocytes, OPCs might also be a growth factor provider for supporting functions of other cell types.

In relation to the notion that OPCs would nourish neighboring cells, our study is consistent with the emerging idea of the concept of neurovascular unit. The neurovascular unit was proposed to understand pathophysiologic responses after stroke and other CNS diseases. This concept emphasized that brain function and dysfunction arise from integrated interactions between neurons, astrocytes, and vascular compartments. Neurovascular responses after injury underlie a transition from acute injury to delayed repair as the brains initiate endogenous angiogenesis and neurogenesis [30], [31], [32], [33]. It is now recognized that in gray matter, angiogenic and neurogenic responses are tightly co-regulated after brain injury, comprising cell-cell signaling within the neurovascular niche [34], [35]. The idea of the neurovascular niche primarily guide research in cell-cell trophic coupling in gray matter. But those interactions are likely to be important in white matter as well. With a guide from the idea of neurovascular unit, we have proposed that an analogous oligovascular niche may also exist and support white matter homeostasis through the cell-cell interaction between cerebral endothelium and oligodendrocytes [20], [36]. Our current findings may provide a novel insight for the roles of oligovascular niche; i.e. in the microenvironment between OPCs and endothelial cells, OPC-derived TGF-β1 helps endothelial cells promote BBB integrity.

Our current study demonstrates the novel roles of OPCs in the CNS vascular system. Nevertheless, there are a few issues that warrant further studies. First, we only focused on the OPC-BBB interaction at post-natal day 0–1, but OPCs also exist in the embryonic stage. How OPCs contribute to the entire temporal spectrum of BBB development, and whether OPCs interact with other glia such as astrocytes and pericytes during BBB maturation should be examined in future studies. Second, OPCs may secrete several soluble factors other than TGF-β1 [20], [27]. Hence, examining the mechanisms of other OPC-endothelium mediators will be very important. Third, we used both male and female pups for preparing OPC cultures. Since we mixed all the brains from one litter during the OPC preparing procedure, our OPC cultures were from approximately 50% male and 50% female OPCs. But potential sex differences in OPC function are important. Previous papers reported that neuron cultures from female rats showed more resistance to stresses than ones from male [37], [38], [39]. But sex differences in OPC function are still mostly unknown. Therefore, future studies should carefully examine the differences between male and female in OPC function on BBB regulation. Finally, our current data of OPC effects on the BBB were obtained in immature systems, and therefore, our findings of endothelium-OPC interaction are limited to the BBB development and maturation period. But our data may show some implications for mature brain pathology/pathophysiology. OPCs comprises about 5% of cells in the adult brain [40] and these populations of OPCs may contribute to white matter homeostasis [41], [42]. Therefore, further effects on BBB function in fully mature brains require more careful analysis in the future. OPCs are known to be vital repair cells for white matter, and BBB dysfunction is an important part of white matter dysfunction in a wide range of CNS diseases [43], [44]. Hence, investigating the mechanisms of OPC-BBB interaction in adult brain may help identify novel therapeutic approaches for protecting vascular homeostasis in white matter [45], [46].

Gliovascular signaling is essential for BBB development and maturation. Our study suggests that besides astrocytes and pericytes, OPCs can surprisingly play an important role in supporting BBB function and homeostasis.
